# RIP mutated ITS genes in populations of *Ophiocordyceps sinensis* and their implications for molecular systematics

**DOI:** 10.1186/s43008-020-00040-0

**Published:** 2020-09-16

**Authors:** Yi Li, Lan Jiang, Ke Wang, Hai-Jun Wu, Rui-Heng Yang, Yu-Jing Yan, Kathryn E. Bushley, David L. Hawksworth, Zujian Wu, Yi-Jian Yao

**Affiliations:** 1 Department of Plant Protection, Fujian Agriculture and Forestry University, Fuzhou, 350002 Fujian China; 2 College of Food Science and Engineering, Yangzhou University, Yangzhou, 225127 Jiangsu China; 3 State Key Laboratory of Mycology, Institute of Microbiology, Chinese Academy of Sciences, Beijing, 100101 China; 4 State Key Laboratory of Bioreactor Engineering, East China University of Science and Technology, Shanghai, 200237 China; 5 Graduate University of Chinese Academy of Sciences, Beijing, 100049 China; 6 Institute of Edible Fungi, Shanghai Academy of Agriculture Sciences, Shanghai, 201403 China; 7 Center for Macroecology, Evolution and Climate, Natural History Museum of Denmark, University of Copenhagen, 100012 Copenhagen, Denmark; 8 Department of Plant and Microbial Biology, University of Minnesota, St Paul, MN 55108 USA; 9 Department of Life Sciences, The Natural History Museum, Cromwell Road, London, SW7 5BD UK; 10 Comparative Plant and Fungal Biology, Royal Botanic Gardens, Kew, Richmond, Surrey, TW9 3DS UK; 11 Jilin Agricultural University, Changchun, 130118 Jilin China; 12 Geography and Environment, University of Southampton, Southampton, SO17 1BJ UK

**Keywords:** Caterpillar fungus, *Cordyceps sinensis*, ITS pseudogene, Haplotype, Phylogeny

## Abstract

Different hypotheses have been proposed to interpret the observed unusual ITS (internal transcribed spacer) sequences in *Ophiocordyceps sinensis*. The coexistence of diverged ITS paralogs in a single genome was previously shown by amplifying the ITS region from mono-ascospore isolates using specific primers designed for different ITS paralog groups. Among those paralogs, are AT-biased ITS sequences which were hypothesized to result from repeat-induced point mutation (RIP). This is a process that detects and mutates repetitive DNA and frequently leads to epigenetic silencing, and these mutations have been interpreted as pseudogenes. Here we investigate the occurrence and frequency of ITS pseudogenes in populations of *O. sinensis* using large-scale sampling, and discusses the implications of ITS pseudogenes for fungal phylogenetic and evolutionary studies. Our results demonstrate a wide distribution of ITS pseudogenes amongst different geographic populations, and indicate how ITS pseudogenes can contribute to the reconstruction of the evolutionary history of the species.

## Introduction

The nuclear ribosome DNA (nrDNA) is considered a classic example of concerted evolution in most eukaryotes (Nei and Rooney 2005; Ganley and Kobayashi 2007; Stage and Eickbush 2007). As one of the most popular components of nrDNA, the internal transcribed spacer (ITS) generally appears to evolve through concerted evolution, and thus has been widely used in species identifications and phylogenetic studies of diverse organisms (e.g. Buckler and Holtsford 1996; Denman et al. 2000; Hřibová et al. 2011; Rouland-Lefevre et al. 2002). It has also been selected as the universal fungal DNA barcode (Schoch et al. 2012). However, intra-genomic ITS polymorphisms, indicating potential escapes from concerted evolution, have long been reported in fungi (e.g. O’Donnell and Cigelnik 1997; Wang and Yao 2005). As one kind of intra-genomic polymorphisms, ITS pseudogenes are one category of such polymorphisms, and these have been reported in a variety of fungal species, including *Colletotrichum graminicola*, *Cordyceps militaris*, *Epichloë* spp., *Laetiporus* spp., *Leptosphaeria maculans*, *Neurospora crassa*, and *Ophiocordyceps sinensis* (Lindner and Banik 2011; Li et al. 2013; Li et al. 2017). Pseudogenes are nonfunctional genes that result from multiple mutations of parental active genes, and which are not usually transcribed.

*Ophiocordyceps sinensis* is a valued traditional Chinese medicine (Pegler et al. 1994), which has attracted increasing scientific attention in recent years and assumed considerable economic significance in some rural economies in the Himalayas (e.g. Shrestha and Bawa 2013; Liang 2018). ITS has been used as an important molecular marker in the identification and genetic analysis of this species (Liu et al. 2001; Kinjo and Zang 2001; Zhang et al. 2009), but dissimilar sequences which could have implications for phylogenetic analyses in this fungus have been obtained. Variations in the ITS among individuals of this species were first identified by Kinjo and Zang (2001), and the polymorphisms have been variously interpreted as genetic divergences (Kinjo and Zang 2001), different genotypes (Zhu et al. 2010), cryptic species (Stensrud et al. 2007), or potentially other species (Xiao et al. 2009).

Li et al. (2013) designed specific primers and amplified different ITS paralogs from mono-ascospore isolates of the same collection, which confirmed the coexistence of different haplotypes in single genomes of this species. Several divergent ITS sequences were highly AT-biased and identified as pseudogenes. A genome defence mechanism, repeat-induced point mutation (RIP), was subsequently shown to have a potential role in creating these pseudogenes (Li et al. 2017). RIPs were first discovered in 5S RNA genes of *Neurospora crassa* (Selker and Stevens 1985). These could result in duplicated sequences greater than ~ 400 bp (or ~ 1 kb for unlinked duplications) and introduce numerous G:C to A:T point mutations (up to ~ 30% of the G:C pair) during a single passage through the sexual cycle of that fungus (Cambareri et al. 1989; Watters et al. 1999). RIP-mutated sequences are frequent targets for DNA methylation, which can cause gene silencing in *Neurospora* (Rountree and Selker 1997), and these have been recognized as pseudogenes. While ITS pseudogenes have already been proved to exist in *O. sinensis* (see above)*,* the distribution and evolutionary history of these ITS pseudogenes within *O. sinensis* populations remained unknown. This new study was designed to investigate the occurrence of RIP-mutated ITS pseudogenes in different population of *O. sinensis* using large-scale sampling and to test the utility of these pseudogenes in reconstructing the evolutionary history of the species.

## Materials and methods

### Taxon sampling and DNA sequencing

**Fig. 1:**
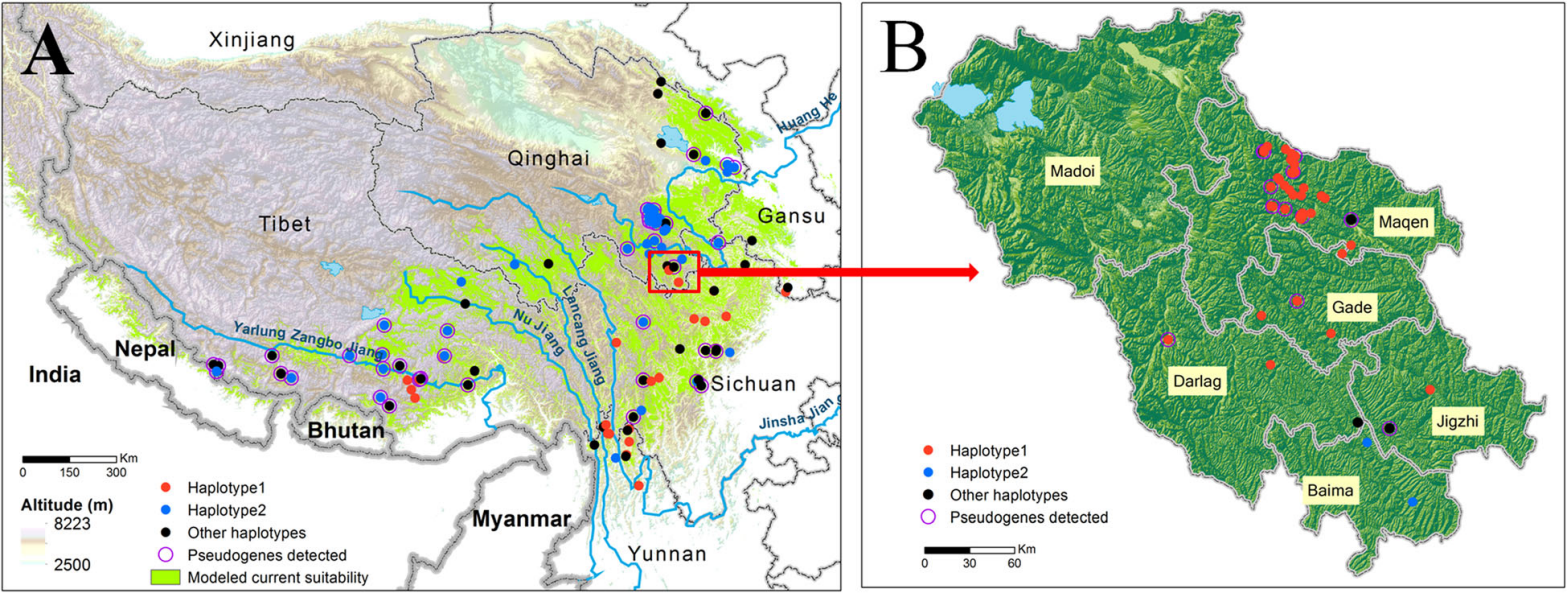
Sampling and detection of ITS pseudogenes in populations of *Ophiocordyceps sinensis* in China (**a**) and Guoluo, Qinghai (**b**), respectively. Red spots indicate the dominant ITS haplotype (haplotype 2), blue spots indicate ITS haplotype (haplotype 1) with the epitype designated by Li et al. (2013) and black spots indicate other haplotypes. Purple cycles indicate the detection of ITS pseudogenic paralogs

A total of 147 individuals were used in this study, including 86, 23, 18, 14 and 4 collected from Qinghai, Tibet, Sichuan, Yunnan and Gansu provinces, respectively, covering nearly the whole collecting regions of *Ophiocordyceps sinensis* in China (Table S1, Fig. 1). To investigate pseudogene occurrences at a finer scale, more extensive sampling was carried out in Guoluo (Qinghai province), especially in Maqên County, where 64 individuals were collected (Table S1). An isolate of *O. emeiensis* (2236), one individual of *O. lanpingensis*, and eight individuals of *O. laojunshanensis* were used as outgroups (Table S1).

The individuals collected were dried with silica gel and one strain (2236) was maintained at 4 °C on Potato Dextrose Agar (PDA) slants. The stocks were transferred to new PDA slants and incubated at 25 °C for 20 d. Fresh mycelia were collected and used for DNA extraction. Total genomic DNA was extracted from either dried inner tissues or cultivated fresh mycelia using the modified CTAB method (Yao et al. 1999). The fungal universal primer pair ITS5/ITS4 (White et al. 1990) was used to amplify functional ITS sequences. Pseudogenes were amplified using 10- to 100-times dilutions of PCR products (amplified with ITS5/ITS4) as the templates and GCF/GCR (Li et al. 2013) as the primers. Amplifications were performed in a 25 μl PCR reaction system which contains 12.5 μl 2 × Taq PCR Master Mix (Tiangen Biotech, China), 0.25 μl of each primer (10 μM) and 1 μl DNA template with a thermal cycler (Applied Biosystems, Foster City, CA). The PCR conditions used were: 2 min at 94 °C; 30 cycles of 94 °C for 30 s, 53 °C (60 °C for the primers GCF/GCR) for 30 s and 72 °C for 45 s; and a final extension at 72 °C for 10 min. PCR products were directly sequenced using the same PCR primers on a capillary sequencer (Applied Biosystems 3730 Analyzer, Foster City, CA) by the Beijing Genomics Institute (Beijing, China). Direct sequencing of ITS pseudogenes for several individuals displayed polymorphisms at a few sites; a cloning method was therefore employed for those samples (Table S2). A 50 μl PCR reaction was used for cloning and sequencing, the PCR products were purified with the Gel Extraction Kit (CWbiotech, Beijing, China), cloned into vectors using the pEASY-T1 Simple Cloning Kit (TransGen Biotech, Beijing, China), and then transformed into Trans1-T1 phage resistant chemically competent cells (TransGen Biotech, Beijing, China). At least one positive clone for each individual was sequenced using primers M13F/SR in the vector. The ambiguous base pairs in obtained sequences were manually edited by checking the chromatograms of both directions before submitting to the alignment.

### Sequence dataset and haplotype analyses

Nine ITS pseudogenes of *O. sinensis*, one ITS sequence of *O*. *emeiensis* (AJ309347) and three ITS sequences of *O*. *lanpingensis* (HQ654775, HQ654776 and HQ654777) were downloaded from GenBank and included in the analyses together with sequences obtained in this study (Table S2). The 18S and 28S nrDNA regions were removed before analyses. Haplotypes of identical sequences were identified and categorized using DAMBE version 4.2.13 (Xia and Xie 2001). The sequence alignment used in this study was deposited in TreeBASE **(**http://purl.org/phylo/treebase/phylows/study/TB2:S24803).

### Length variations, GC contents, sequence divergence levels, and RIP analyses

The lengths and GC contents of the ITS1–5.8S-ITS2 regions were calculated using BioEdit version 7.0.9.0 (Hall 1999). Sequence divergence levels (average *p*-distance) of functional ITS sequences and pseudogenes were analyzed by MEGA5 (Tamura et al. 2011). Pairwise deletion was used for missing data. RIP analyses were performed using RIPCAL (Hane and Oliver 2008). Sequences with the highest GC content were used as consensus.

### ITS pseudogene identification and mutation analyses

The ITS pseudogenes in *O. sinensis* were characterized by lower GC contents, different secondary structures, and higher minimum free energies of the 5.8S rRNA transcripts compared with their functional counterparts, due to a number of random G:C to A:T transition mutations introduced by RIP (Li et al. 2013, 2017). The G:C to A:T transition mutations in ITS sequences were analyzed and used as the main criteria for ITS pseudogene identification. Sequences amplified with primer pair GCF/GCR were aligned and compared with the functional counterparts (amplified by ITS5/ITS4 from the same individuals). Those sequences in which G:C to A:T mutations occurred compared to functional ones were identified as pseudogenes. C → T, G → A and other mutation types were counted for different ITS regions (ITS1, 5.8S and ITS2) of the identified pseudogenes using functional counterparts from the same individuals as references.

### Phylogenetic analyses

Sequences were aligned with ClustalW (Thompson et al. 1994) and the alignments were manually refined with BioEdit. The boundaries of the ITS1, 5.8S, and ITS2 regions were determined according to previously published ITS sequences, and the 18S and 28S regions were removed from the alignments. ITS phylogenies were constructed using Maximum Likelihood (ML) and Bayesian Inference (BI). ML analyses were conducted with RAxML version 7.2.6 (Stamatakis 2006) to obtain the best ML tree as well as to determine bootstrap supports (BS). The best-fit nucleotide substitution model GTR + G was determined using MrModeltest version 2.2 (Nylander 2004) based on Akaike Information Criterion (Akaike 1974). Node supports were assessed with 1000 bootstrap replicates. Bayesian analyses were implemented with MrBayes v3.2.6 (Ronquist et al. 2012) using the same substitution model. Two independent analyses of two parallel runs and four chains were carried out for 5,000,000 generations. Trees were sampled every 1000 generations, the first 20% of the trees being discarded as burn in. A consensus tree and posterior probabilities (PP) were calculated. *Ophiocordyceps emeiensis*, *O*. *lanpingensis*, and *O*. *laojunshanensis* were used as outgroup taxa. Different phylogenetic analyses were performed independently for the three ITS datasets, i.e. functional and pseudogenes separately, and the combined dataset.

## Results

### Amplification, identification and geographic distribution of ITS pseudogenes

**Fig. 2:**
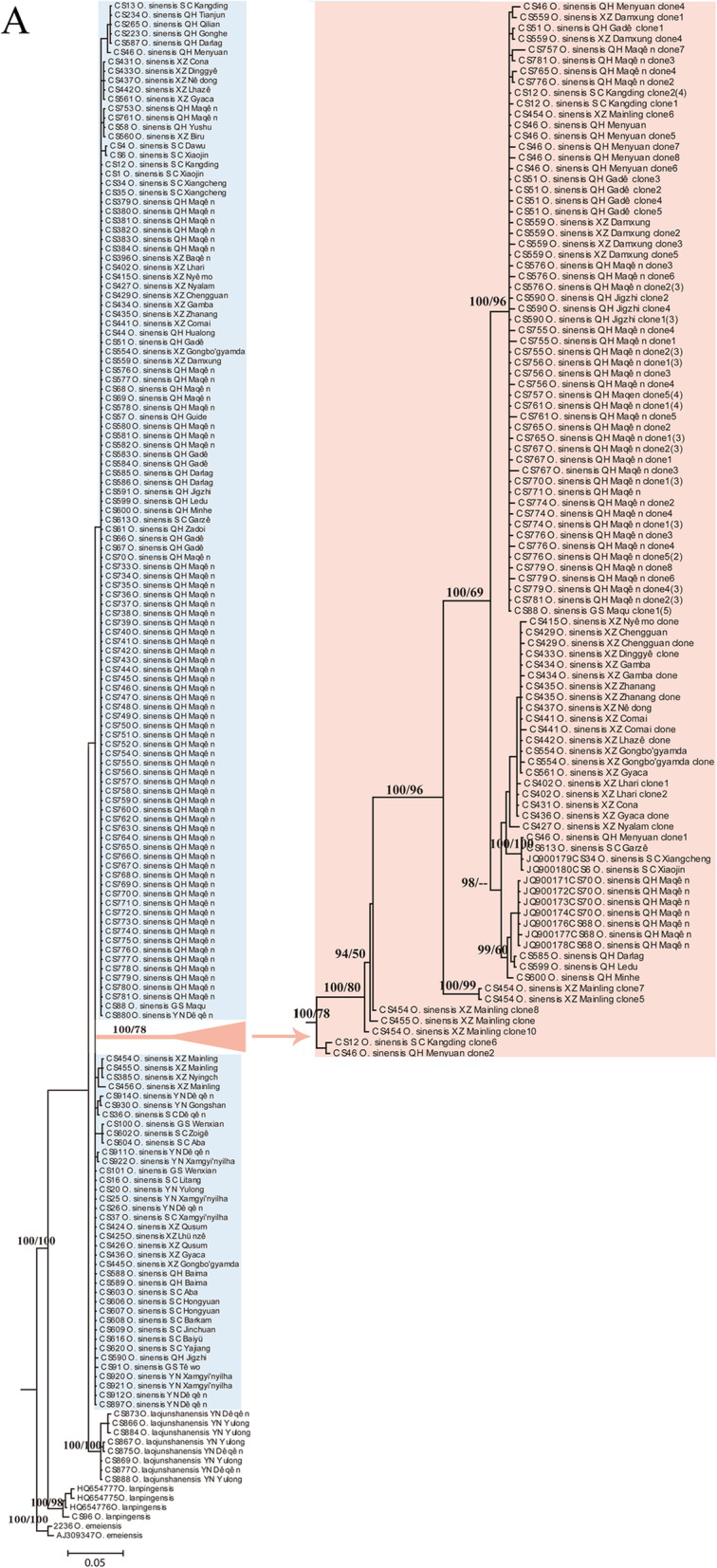

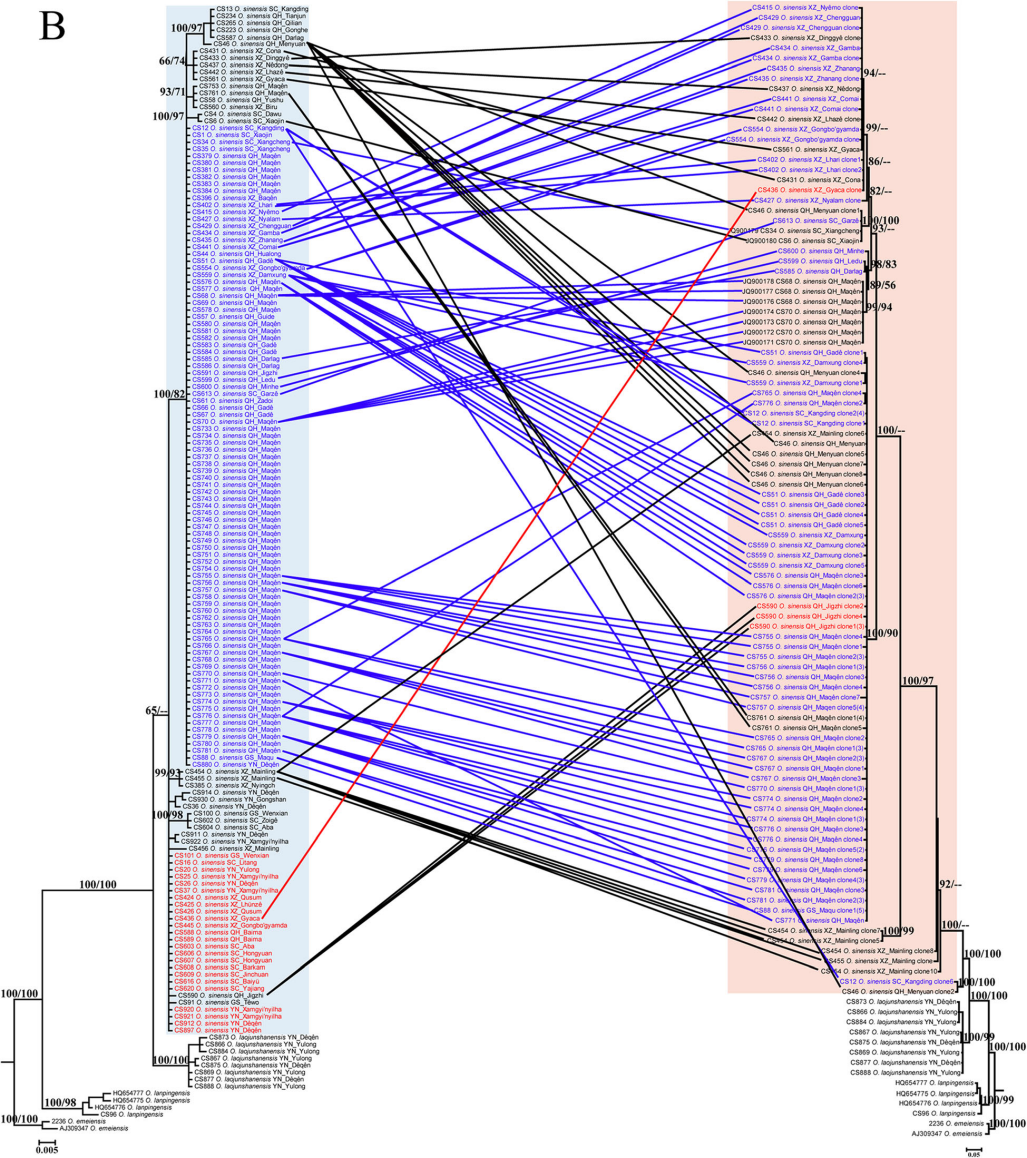
*Continued*

A total of 268 ITS sequences were obtained from the 147 individuals of *O. sinensis*, among which 123 were identified as pseudogenes (Table S2). Of those pseudogenes, 15 were obtained by direct PCR and sequencing, and the others (108) by cloning and sequencing. ITS pseudogenes were detected from ~ 29% (43 out of 147) of the tested individuals and were found to be widely distributed in populations from Qinghai, Tibet, Sichuan, and Gansu provinces, but were not observed in any from Yunnan Province (Fig. 1, Table S2). The occurrence rate of ITS pseudogenes was ~ 24% (18 out of 76) in Guoluo, Qinghai, and ~ 26% (17 out of 66) in Maqên, Guoluo, respectively (Table S1). According to the DAMBE analyses, the 130 ITS pseudogenes (nine from GenBank) represented 57 haplotypes (unique sequences), and the 147 functional genes represented 17 haplotypes. The number of ITS pseudogene haplotypes varied among the 43 individuals in which they were detected, ranging from one (e.g. CS431, CS433, CS437) to eight (CS46) haplotypes per individual (Fig. 2b).

### Length variations, GC contents and sequence divergences

The length of the functional ITS sequences was 562 bp in most individuals of *O. sinensis*. The exceptions were two (CS4 and CS6) collected in Dawu and Xiaojin County, Sichuan Province; in these an 8-bp motif 5′-CGCCCCGG-3′ in ITS1 was found to be missing (Table 1). Regions of 95 bp in ITS1 and 50 bp in ITS2 were not obtained for the ITS pseudogenes of *O. sinensis* because the PCR primers were located inside the two spacer regions. The lengths of the amplified region of the ITS pseudogenes were generally 417 bp, the same as the corresponding region in functional counterparts (Table S2).

**Table 1:** ITS1–5.8S-ITS2 Length variation, GC content and *p*-distance analyses of *O. sinensis* and related species studied

Groups	Length variation	Average GC content	Average ***p***-distance
*O. emeiensis*	568 bp, 569 bp	59.72%	0.007
*O. lanpingensis*	576 bp, 577 bp	60.19%	0.009
*O. laojunshanensis*	562 bp, 564 bp	62.57%	0.005
*O. sinensis* (functional)	562 bp, 554 bp	63.13%	0.006
*O. sinensis* (pseudogene)	417 bp	51.33%	0.044

The GC contents of ITS functional sequences varied amongst *O*. *emeiensis*, *O*. *lanpingensis*, *O*. *laojunshanensis* and *O. sinensis*, ranging from 59.58 to 59.86% (avg. 59.72%), 59.45 to 60.38% (avg. 60.19%), 62.46 to 62.77% (avg. 62.57%), and 59.79 to 63.70% (avg. 63.13%), respectively (Table 1). The GC contents of the ITS pseudogenes in *O. sinensis* were lower than those in the functional counterparts, ranging from 44.36 to 61.39% (avg. 51.33%) (Table S2). The significantly lower GC contents resulted from an accumulation of G:C to A:T transition mutations, identified in the RIP analyses.

The average *p*-distances for *O*. *emeiensis*, *O*. *lanpingensis*, *O*. *laojunshanensis* and ITS functional sequences of *O. sinensis* were 0.007, 0.009, 0.005, and 0.006, respectively. However, the ITS pseudogenes of *O. sinensis* had an average *p*-distance of 0.044, significantly greater than functional genes of the species and the other closely related species examined (Table 1). This indicates that the pseudogenes are more divergent than their functional counterparts.

### RIP and mutation analyses

The RIPCAL analyses showed that RIP (characterized by a high rate of G:C to A:T transitions) occurred in all the ITS pseudogenes (Fig. S1). CpA → TpA, TpG → TpA dinucleotides were found to be more frequently mutated than the other dinucleotides (Fig. S1). The numbers of RIP mutations were generally close to each other in nearly all the ITS pseudogenes, and varied in only a few cases (e.g. in some clones of CS454, CS455 and CS46) in which RIP mutations occurred in less than half of the ITS region in those sequences (Fig. S1). The average frequencies of C → T and G → A transition mutations for ITS pseudogenes in *O. sinensis* were 7.07 and 6.33%, respectively, while the frequency of all the other mutation types accounted for less than 0.6%. Mutations occurred in both the coding (5.8S) and two non-coding spacers (ITS1 and ITS2) (Table S3).

### Phylogenetic analyses

A total of 147 functional ITS sequences and 98 pseudogenes of *O. sinensis* were included in the phylogenetic analyses. Fourteen sequences from three closely related species, i.e. two from *O*. *emeiensis*, four from *O*. *lanpingensis,* and eight from *O*. *laojunshanensis* were used as outgroups. The consensus tree generated from the Bayesian analyses had a similar topology to the best-scoring ML tree (Fig. 2). Analyses using the three different datasets (functional ITS, pseudogenes, and the combined dataset) all supported the monophylies of the three outgroup species with high support values (BS = 98–100%, PP = 1.00, Fig. 2). A sister relationship between *O*. *laojunshanensis* and *O. sinensis* was supported using functional genes and pseudogenes alone (BS = 100%, PP = 1.00, Fig. 2b), but not by the combined dataset (Fig. 2a). *Ophiocordyceps laojunshanensis* was displayed as the closest relative of *O. sinensis*, followed by *O*. *lanpingensis* and then *O*. *emeiensis*. The monophyly of *O. sinensis* was highly supported (BS = 100%, PP = 1.00) by the pseudogenes, but was only weakly supported using either functional sequences or the combined dataset in both ML and Bayesian analyses (Fig. 2). The monophyly of the ITS pseudogenes was strongly supported by the ML (BS = 100%) and Bayesian (PP = 1.00) analyses using pseudogenes alone or in the combined dataset (Fig. 2).

## Discussion

The ITS phylogeny revealed *O. laojunshanensis* as the closest known relative of *O. sinensis*, and individuals from northwestern Yunnan were more closely related to that species than those from other areas (Fig. 2). As *O. laojunshanensis* appears to be confined to northwestern Yunnan and also occurs in mountainous areas at high elevations, *O. sinensis* is most likely to have originated in northwestern Yunnan by sympatric speciation and not from Lingzhi, Tibet as suggested by Zhang et al. (2009).

The coexistence of ITS functional genes and pseudogenes within single individuals was confirmed either by PCR amplification and sequencing or by cloning and subsequent sequencing. ITS pseudogenes were detected from nearly one-third of the individuals examined, covering a wide geographical range in regions of Qinghai, Tibet, Sichuan, and Gansu Provinces. It was surprising that no pseudogenes were detected from populations in Yunnan Province, even though 14 individuals from there were tested.

The nuclear genome of *O. sinensis* experienced a large expansion in genome size due to an expanded repeat content compared to other closely related hypocrealean species (Hu et al. 2013; Li et al. 2016a; Xia et al. 2017). Given that RIP was shown to induce rRNA pseudogenes in filamentous ascomycetes (Li et al. 2017), including in the genome of *O. sinensis* (Hu et al. 2013), it is reasonable to hypothesize that the occurrence of RIP induced ITS pseudogenes accompanied this species’ genome expansion and speciation. This suggests that the ITS pseudogenes mainly arose after, but not prior to, the origin of *O. sinensis*, and the genomes of *O. laojunshanensis* and other outgroup taxa have not been found to have expanded in the same way (data not shown). However, evidence from genome analyses of these outgroup taxa together with additional genomes of *O. sinensis*, especially from northwestern Yunnan Province, are needed to test whether RIP induced ITS pseudogenes really corresponded with the divergence of this species.

The RIP-mediated mechanism of mutation is only known to occur during sexual reproduction (Freitag et al. 2002). As *O. sinensis* is one of the only known homothallic species in *Ophiocordycipitaceae*, allowing individual strains to mate with themselves (Bushley et al. 2013; Hu et al. 2013), it is possible that the homothallic life-style may have increased the likelihood and frequency of sex in *O. sinensis*, leading to increased RIP in this species. It should also be noted that RIP may not be confined to the ITS region but could extend to the entire nrDNA operon (Li et al. 2017) and also might be able to impact other single-copy sequences adjacent to duplicated sequences (Irelan et al. 1994; Li 2013a).

Haplotype analyses revealed that different ITS pseudogenes could exist in single individuals of *O. sinensis*, that individuals possessing the same functional ITS haplotypes could have the same (e.g. in CS51 and CS559) or different (e.g. in CS12 and CS51) pseudogene ITS haplotypes, and that individuals with different functional haplotypes might share the same pseudogenic ITS haplotypes (as in CS402, CS431 and CS436). Since the divergences in functional copies of the ITS were assumed to be correlated with species diversification in *O. sinensis* (Jiao 2010; Zhang et al. 2009), our results suggest that ITS pseudogenes could have emerged either before or after a new functional ITS haplotype was generated (by random mutation) and homogenized (through concerted evolution). This pattern could be explained by an RIP mechanism, responsible for nrDNA pseudogene formation, in which mutation occurred randomly. The RIP mutated pseudogenes would not face the same evolutionary constraints as functional genes but could be preserved through generations for extended periods of time (Li et al. 2017). If the pseudogene haplotypes arose by a shared RIP mutation, individuals sharing the same pseudogenic ITS haplotypes should have the same ancestors, and RIP mutated pseudogenes could serve as good candidate markers to reconstruct the evolution, diversification and dispersal history of *O. sinensis*.

The ITS region has been widely used in species identifications and fungal phylogenies (e.g. James et al. 2006; Stensrud et al. 2007), and is now accepted as the universal DNA barcode to be adopted in all fungal groups (Schoch et al. 2012) and is also being applied to quantify and characterize environmental fungal diversity (e.g. O’Brien et al. 2005; Buée et al. 2009). The occurrence of ITS pseudogenes has implications for both types of application. Their use in phylogenetic reconstruction has been discussed since their discovery, and they have been used as outgroups in phylogenetic analyses (Buckler and Holtsford 1996) when functional counterparts from closely related species were not available (Razafimandimbison et al. 2004). However, ITS pseudogenes may result in long-branch attraction causing phylogenetic errors (Buckler et al. 1997; Wei et al. 2003; Won and Renner 2005) and may not always cluster with the conspecific functional sequences (e.g. Muir et al. 2001). In this study, the ITS sequences from *O. sinensis* formed a distinct monophyletic clade in ML and BI phylogenetic analyses regardless of whether functional ITS genes, ITS pseudogenes, or the combined dataset were used – although the clade was not well supported in some cases. These results suggest that both functional and pseudogene ITS sequences can accurately distinguish this species from other closely related *Ophiocordyceps* species. However, if distance based methods such as Neighbour Joining were used, the distinct clustering of functional and pseudogene clades could be disturbed, and sequences from the sister species *O. laojunshanensis* were merged (Figure S2). The clustering pattern revealed in a distance based phylogeny would largely depend on the RIP mutation number (Li et al. 2017). There are other cases where pseudogenes were not clustered with conspecific functional sequences (e.g. Lindner and Banik 2011), but ITS pseudogenes are not likely be a critical threat to fungal taxonomy, phylogenetics and related applications as they are rarely detected using standard PCR protocols and can be technically identified (Bailey et al. 2003; Hartmann et al. 2001; Muir et al. 2001; Xiao et al. 2010). Nonetheless, the prevalence of ITS pseudogenes in this species suggests that ITS functional sequences rather than pseudogenes should be used in evolutionary phylogenetic studies, and that the recognition of rDNA pseudogenes prior to such analyses is required. Caution is therefore needed when using ITS as a sole basis for fungal species identification in *O. sinensis* (Kinjo and Zang 2001; Stensrud et al. 2007; Xiao et al. 2009; Zhu et al. 2010). Pseudogenes are of particular concern in relation to studies of fungal community analyses in environmental samples using high-throughput sequencing technologies; divergent rDNA pseudogenes would be unknowingly sequenced and most probably recognized as other species, so leading to overestimates of the fungal species diversity. It is clear that effective methods for rDNA pseudogene identification in sampless are an urgent need for both systematic and environmental mycology.

*Ophiocordyceps laojunshanensis* clustered with *O. sinensis* in a seven gene phylogenetic analysis and was thus considered as a synonym by Li (2013b), in contradiction to our study. This discrepancy was most probably attributable to insufficient and imbalanced taxon sampling; ITS sequences of *O. laojunshanensis* cluster with those of *O. sinensis* if fewer sequences of the former are used, but can be separated with a higher support value if more sequences are included as revealed by a parallel phylogenetic analysis (data not shown). The two species can in any case be distinguished morphologically by characters of the stromata and perithecia (Chen et al. 2011), especially when mature.

Sequences from type material will facilitate the discrimination of these two species and contribute to fixing the species concept of *O. sinensis*. We attempted to amplify ITS sequences from two isotypes of the basionym of *O. sinensis, Sphaeria sinensis,* now preserved in the Kew Fungarium (K(M) 166,697 and K(M) 32,163), but without success. In order to precisely fix the application of the name for the future, we plan to formally designate a sequenced specimen from one of the core regions in which the species is found (CS769, from Maqên, Guoluo, Qinghai; HMAS 281396) as epitype in a forthcoming publication, along with details of the molecular studies attempted on the isotypes, and the formal selection of one of the isotypes as lectotype for the name. Ninety-one individuals, including 1 from Gansu, 74 from Qinghai, 10 from Tibet, 1 from Yunnan, and 5 from Sichuan Provinces share the identical functional ITS sequences (haplotype) with the epitype we are to designate (Fig. 2).

ITS pseudogenes were less frequently observed in individuals from the edge of the Tibetan Plateau than those from the central part of the Plateau (Fig. 1). This observation also supports the hypothesis that ITS pseudogenes arose after, not prior to, the speciation of *O. sinensis*. We speculate that, if northwestern Yunnan was indeed the centre of origin of *O. sinensis*, pseudogenes may have accumulated along with the species diversification as it spread across the Plateau.

## Conclusions

The first discovery of pseudogenic ITS sequences in *Ophiocordyceps sinensis* was in 2001. Since that time, different hypotheses have been proposed to explain these, including representing cryptic species, distinct species, or different genotypes. This study proved that ITS functional genes and pseudogenes coexist in single individuals by PCR amplification using mono-ascospore isolates. Our large scale sampling demonstrated that ITS pseudogenes were widely detected simultaneously along with functional ITS genes in many individuals gathered from diverse populations and localities. ITS pseudogenes, whether from mono-ascospore isolates, tissue isolates, or from dried specimens, all clustered in a highly supported clade, together with the functional ITS clade of *O. sinensis*, suggesting that in addition to functional ITS sequences, ITS pseudogenes can accurately circumscribe this species and so may not pose a great threat to the use of the ITS region as a universal barcode for its identification. The patterns revealed by our phylogenetic analyses support the hypothesis that ITS pseudogenes evolved through RIP mutation throughout the evolutionary history of *O. sinensis*. They also provide evidence that ITS pseudogenes occur frequently and have a wider distribution in populations of the species than previously appreciated, and are not restricted to certain individuals or production areas.

## Supplementary information

**Additional file 1: Table S1**. Voucher information and ITS pseudogene amplification. **Table S2**. Length variation and GC content of ITS functional and pseudogenic sequences used in this study. **Table S3**. Mutation analyses of ITS pseudogenes.

**Additional file 2: Fig. S1**. RIP mutation in ITS sequences of *Ophiocordycepssinensis* shown as RIPCAL output. Functional sequences with the highest GC content, i.e., CS223 and CS234, were defined as consensus. Black, invariant nucleotide; white, gap; red, CpA ↔ TpA or TpG ↔ TpA mutations; dark blue, CpC ↔ TpC or GpG ↔ GpA mutations; green, CpG ↔ TpG or CpG ↔ CpA mutations; pale blue, CpT ↔ TpT or ApG ↔ ApA mutations.

**Additional file 3: Fig. S2**. Neighbor joining phylogenetic tree inferred from the combined dataset of ITS genes and pseudogenes.

## Data Availability

The datasets analyzed during the current study are available from the corresponding author on reasonable request.

## Abbreviations

BI: Bayesian Inference
BS: Bootstrap support
CTAB: Cetyltrimethylammonium Bromide
ITS: Internal transcribed spacer
ML: Maximum likelihood
nrDNA: Nuclear ribosome DNA
PCR: Polymerase chain reaction
PDA: Potato Dextrose Agar
PP: Posterior probabilities
RIP: Repeat-induced point mutation
