# Supplementary figures and images for: RIP mutated ITS genes in populations of Ophiocordyceps sinensis and their implications for molecular systematics
Source: IMA Fungus. 2020 Sep 16;11:18. doi: 10.1186/s43008-020-00040-0 (PMC7493409; doi:10.1186/s43008-020-00040-0)

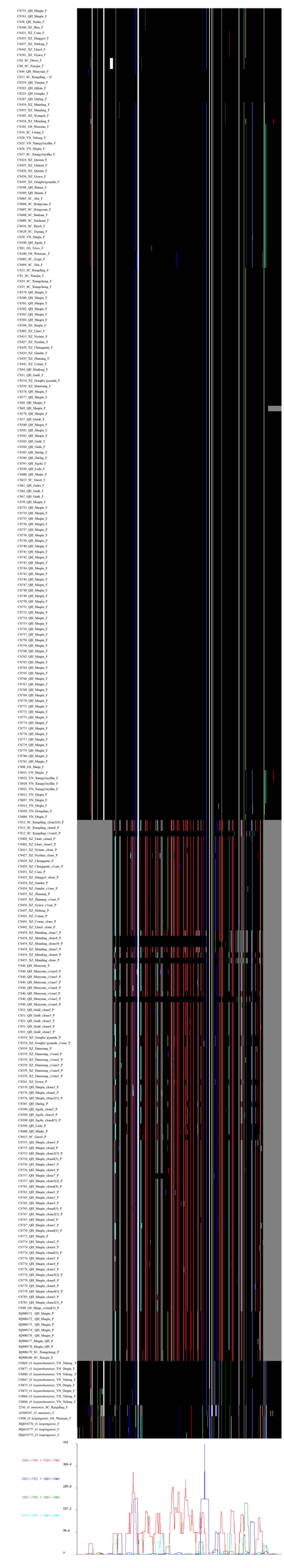

Supplement: Supplementary file 2 — Additional file 2: Fig. S1. RIP mutation in ITS sequences of Ophiocordycepssinensis shown as RIPCAL output. Functional sequences with the highest GC content, i.e., CS223 and CS234, were defined as consensus. Black, invariant nucleotide; white, gap; red, CpA ↔ TpA or TpG ↔ TpA mutations; dark blue, CpC ↔ TpC or GpG ↔ GpA mutations; green, CpG ↔ TpG or CpG ↔ CpA mutations; pale blue, CpT ↔ TpT or ApG ↔ ApA mutations. [file 43008_2020_40_MOESM2_ESM.jpg]

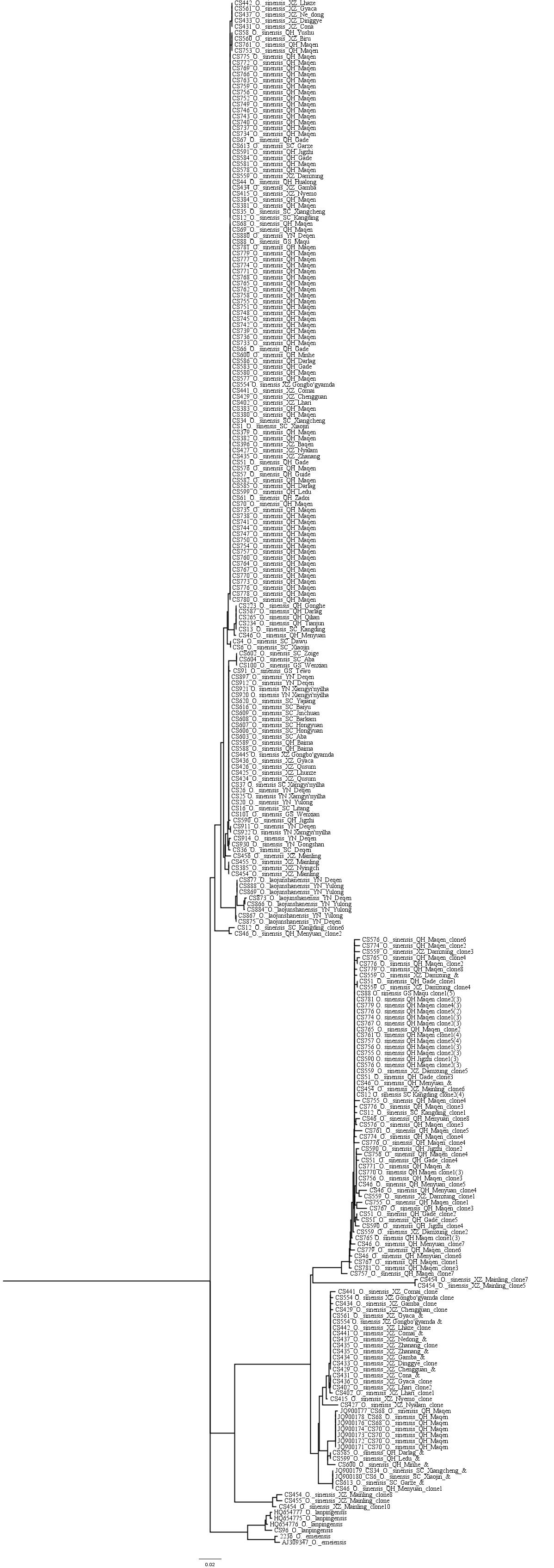

Supplement: Supplementary file 3 — Additional file 3: Fig. S2. Neighbor joining phylogenetic tree inferred from the combined dataset of ITS genes and pseudogenes. [file 43008_2020_40_MOESM3_ESM.jpg]
